# Deep Learning Based Homomorphic Secure Search-Able Encryption for Keyword Search in Blockchain Healthcare System: A Novel Approach to Cryptography

**DOI:** 10.3390/s22020528

**Published:** 2022-01-11

**Authors:** Aitizaz Ali, Muhammad Fermi Pasha, Jehad Ali, Ong Huey Fang, Mehedi Masud, Anca Delia Jurcut, Mohammed A. Alzain

**Affiliations:** 1 Department of Software Systems and Cyber-Security, School of IT, Monash University, Jalan Lagoon Selatan, Bandar Sunway, Subang Jaya 47500, Malaysia; aitizaz.ali@monash.edu (A.A.); muhammad.fermipasha@monash.edu (M.F.P.); Ong.hueyfang@monash.edu (O.H.F.); 2 Department of Computer Engineering, Ajou University, Suwon 16499, Korea; 3 Department of AI Convergence Network, Ajou University, Suwon 16499, Korea; 4 Department of Computer Science, College of Computers and Information Technology, Taif University, P.O. Box 11099, Taif 21944, Saudi Arabia; mmasud@tu.edu.sa; 5 School of Computer Science, University College Dublin, Belfield 4, D04 V1W8 Dublin, Ireland; anca.jurcut@ucd.ie; 6 Department of Information Technology, College of Computers and Information Technology, Taif University, P.O. Box 11099, Taif 21944, Saudi Arabia; m.alzain@tu.edu.sa

**Keywords:** security, blockchain, privacy, homomorphic encryption, deep learning, access control, smart contracts

## Abstract

Due to the value and importance of patient health records (PHR), security is the most critical feature of encryption over the Internet. Users that perform keyword searches to gain access to the PHR stored in the database are more susceptible to security risks. Although a blockchain-based healthcare system can guarantee security, present schemes have several flaws. Existing techniques have concentrated exclusively on data storage and have utilized blockchain as a storage database. In this research, we developed a unique deep-learning-based secure search-able blockchain as a distributed database using homomorphic encryption to enable users to securely access data via search. Our suggested study will increasingly include secure key revocation and update policies. An IoT dataset was used in this research to evaluate our suggested access control strategies and compare them to benchmark models. The proposed algorithms are implemented using smart contracts in the hyperledger tool. The suggested strategy is evaluated in comparison to existing ones. Our suggested approach significantly improves security, anonymity, and monitoring of user behavior, resulting in a more efficient blockchain-based IoT system as compared to benchmark models.

## 1. Introduction

A patient health record (PHR) is the basic and important information related to a patient’s history and related details. The digital healthcare system is considered as the platform for transferring and receiving patient health records. However, the existing digital healthcare systems rely on centralized servers, which are more prone to security breaches. Hence, the simplest solution is to integrate the digital healthcare system to blockchain technology due to its wide variety of applications and improved security. More importantly, blockchain provides peer-to-peer (P2P) and decentralized network systems. In general, blockchains can be classified into three different categories, namely, private, public, and consortium blockchains [1]. It is a permissioned and consortium managed blockchain, which means all peers are known to each other in the network. This provides trust and security to all the parties involved. Hyperledger fabric is not domain specific, and it supports Java, Go, Node.js, etc., for creating contracts and networks applications [2].

In industrial applications, healthcare is considered to be a vital field. In addition to a classic medical diagnosis, patients’ body parameters, comprised of heart rate, blood sugar level, electroencephalogram, and additional vital biomedical signals can be monitored by integrating numerous medical sensing devices for diagnosis [3], as well as health quality improvement. Medical diagnosis, biomedical research, and policy making sharing can provide easiness in sharing and secure access. The Internet of Things (IoT) has been proposed to enhance quality of the industries, break regional limitations to achieve remote monitoring, perform autonomous production and deliver real-time information to the end users. For instance, the healthcare industry can often take advantage of the IoT, where industrial sensors and actuators are used as wearable devices to collect users’ physiological data, such as blood pressure, electrocardiogram (ECG), temperature, and so on. These physiological data are usually sent to the nearby devices or servers of the user to carry out further data collection, aggregation, and then sent for diagnosis and input to a industrial provider using an open channel, i.e., the Internet. However, due to the round-the-clock networking of nodes in this IoT network, it is vulnerable to various security issues, such as message tampering, eavesdropping, and denial of service (DoS) attacks. In the industrial industry, this raises major security issues as the misuse of data can result in incorrect diagnosis and can cause life-threatening scenarios for the patients under observation. For instance, a clinician may want to access the medical records of a patient located in different hospitals databases in order to decide for the best treatment of a patient. Moreover, this market can bring a huge and positive impact on the economy. In a nutshell, the digital healthcare systems’ data sharing and trust are key factors for success, and deficiency could result in distrust among patients towards the e-healthcare market [4].

There exist several searchable encryption (SE) methods to provide solution to the problems as mentioned above, but they are not as efficient regarding flexibility and anonymity. SE can be categorized into different types based on several parameters such as single write (SW), multiple write (MW) single read (SR), and multiple read (MR) strategies. However, all SE approaches are not as efficient when deploying to the cloud or server-based architecture systems. One of the most promising and secure approaches to solve these issues is secure searchable encryption (SSE), which enables the users to encrypt the data on their own side without the involvement of a third party. Secure searchable encryption can be divided into two groups named Asymmetric SSE and Symmetric SSE [4]. Our proposed extended secure searchable encryption (ESSE) is based on the motivation from [5]. The authors proposed the idea of Obvious cross Tag (OXT) searchable mechanism. The idea of OXT is to distribute all master keys among the users in order to take more advantages of the protocol. The problem with OXT is the key loss or collusion attack, which make it more prone to vulnerabilities. Our proposed approach is more resilient to active collusion attack and key loss situations. Moreover, our proposed method is flexible enough to be applied to different platform such as social media, fog computing, and other IoT-based applications [6].

In this research paper we have proposed extended multi-user-extended SSE which support the participants to query securely against desired keyword search in the distributed ledger. The patient encrypts the data at the beginning and uploads it to the blockchain. Our research method provides facility to the data owner once the data owner completed the encryption, it will not be necessary to be involved in other processes until he or she needs policy revocation or deletion.

As known for its flexibility and accessibility, cloud computing is a multi-functional platform for data storage. However, data outsourced to the cloud might be considered insecure because the data owner’s control over the data is limited, which potentially poses more security issues. Similarly, security issues become the primary concern when it comes to the healthcare industry. Most of the current healthcare data and patient records are stored in the cloud, thus, dealing with medical records is a concern which is widely shared. Digital health records including sensitive patient data are seen as some of the most valuable data, however, it is also vulnerable due to the cloud-based platform, which gives the unethical third parties more chances to access or steal the data due to the market value [7]. Although advancement has been made in access control schemes and frameworks, however, issues still exist in the proposed schemes. These include the absence of measuring granularity in authorizing [8], reliance on identity, and role or purpose-based access control schemes [9]. The existing access control system only relies on identity, or role-based and attribute-based methods. Through a series of comparative analysis, it has been observed that the most appropriate access control strategy among the existing solutions is ABE [10]. The Public-Key encryption does not overcome the security aspects of the attribute-based encryption. Consequently, in the proposed solution, we have used Attribute Based Signature (ABS), since it guarantees anonymity as well as unforgeability to the signer [11,12]. The aim of our proposed strategy is to secure the Personal Health Record (PHR) leveraging homomorphic encryption and fine-grained access control without any security loss or threat. To achieve our goals, we propose a novel protocol based on data sharing via combining two latest attribute based cryptography schemes and homomorphic encryption with an ABAC model using hyperledger fabric. Furthermore, we have provided a detailed comparison based evaluation of our scheme with several state-of-the-art schemes. The critical difference of the proposed scheme and the traditional the application of the computational trust-based value. The advantages of trust evaluation includes:

1. Developing a novel lightweight consensus mechanism by combining with the BFT protocol;
2. Measuring the trustworthiness of the user and presumers before creating smart contracts and before initiating interactions among several parties;
3. Moreover, it also helps in the accountability of the privacy and consent violation;
4. In addition, it helps to check the integrity before adding them to the genesis because the existing ABAC and RBAC systems have low efficiency, and these are not based on learning-based methods [13];
5. Finally, we achieve more efficiency and security.

Our proposed approach will examine the parameters chosen, including user behavior, attributes, trust, unauthorized request, forbidden request, and range of specification. Users will be divided into different categories based upon the trust value such very low, low, unknown, moderate, high, and very high trusted users. A threshold value will be set, i.e., if a user meets the threshold value and also meets the policy, then access will be granted.

In this section, we provide details and preliminaries in order to proof the application of AI in blockchain and homomorphic encryption. Moreover, our proposed system provides trust to several parties and individuals withing the consortium’s blockchain. Our main motivation is to design a blockchain based secure searchable framework that uses deep learning techniques in order to build a trust. Finally, our goal is to build a generic DL-based secure searchable model that can support several different types of AI scenarios and different types of datasets. In order to obtain the scalability and reliability in cloud-based healthcare data sharing schemes illustrated in [14], the schemes have been designed in the literature by implementing the encryption and operation anonymization. Moreover, participants are always curious to transfer their private and sensitive data to the cloud due to the potential risks [15]. For example, the medical industry can also benefit from the industrial IoT (IIoT), where industrial sensors and actuators are used as wearable devices to collect users physiological data, such as blood pressure, electrocardiogram (ECG), temperature, and so on. These physiological data are usually sent to the nearby devices or servers of the user to carry out further data collection, aggregation, and then sent for diagnosis and input to an industrial provider using an open channel, i.e., the Internet. However, due to the round-the-clock networking of nodes in this IoT network, it is vulnerable to various security issues, such as message tampering, eavesdropping, and denial of service (DoS) attacks. In the industrial industry, this raises major security issues as the misuse of data can result in the incorrect diagnosis and can cause life-threatening scenarios for the patients under observation. Privacy issues are often related to compromising sensitive information by using active and passive attacks. In active attacks, the adversary aims to obtain access, infer, and/or alter private data. Recently, several research works related to data privacy protection and identification of cyber-attacks particularly using cloud and edge computing integrated with blockchain are proposed to tackle the above issues. However, it should be noted that there still exist demerits in the system. The major challenges include designing a privacy-preservation scheme to protect the sensitive data transactions against being accessed by unauthorized users [16]. Moreover, ensuring a secured authentication data transfer scheme and maintaining data integrity when communicating the data over an IoT network is a challenging issue. Second, designing an adaptable security mechanism that can efficiently distinguish normal and attack instances in IoT is also a challenging issue. As such, the IoT network comprises various interconnected medical sensors, actuators, and machines (e.g., VMs and platforms), located at multiple locations [17]. Third, developing a new framework for deploying blockchain and deep learning techniques in current cloud-edge-assisted industrial systems is strenuous as such frameworks often face issues related to scalability. Moreover, due to the different computing powers of the participating edge nodes, it is infeasible to store the complete block in the edge networks [18]. The IIoT has been proposed to dramatically enhance quality of traditional industries, break regional limitations to achieve remote monitoring, perform autonomous production and provides real time information to users [19]. For example, the medical industry can also benefit from the IoT where industrial sensors and actuators are used as wearable devices to collect users’ physiological data, such as blood pressure, electrocardiogram (ECG), temperature, and so on. This physiological data are usually sent to the nearby devices or servers of the user to carry out further data collection, aggregation, and then sent for diagnosis and input to a industrial provider using an open channel, i.e., the Internet. However, due to the round-the-clock networking of nodes in this IoT network, it is vulnerable to various security issues, such as message tampering, eavesdropping, and denial of-service attacks [20]. In the healthcare industry, this raises major security issues as the misuse of data can result in the incorrect diagnosis and can cause life-threatening scenarios for the patients under observation [21]. Moreover, data stored on a blockchain are highly reliable and available through replication.

In this subsection, we enumerate the contributions of our proposed methodology, which are as follows:

1. A detailed literature review of the state-of-the-art schemes of patient and participants detection based on encryption ad security algorithm;
2. Novel cross-domain and access control policies are proposed using homomorphic encryption;
3. Moreover, a new privacy-preservation and intrusion detection framework is designed by using blockchain and deep learning technique that enables two-level privacy;
4. In first level of data security and privacy preservation, a blockchain and smart contract scheme is suggested to enable immutable data exchange and to avoid data poisoning attacks;
5. We have achieved an optimum security and anonymous keyword search in the hyperledger fabric framework;
6. Our proposed research method provides an alternative private key in case a key is lost;
7. We have achieved the efficiency as compared to the existing methods as these methods exhibit more communication and encryption cost as these methods need to encrypt the data. Our proposed scheme provides an efficient solution to the users.

The remaining structure of the paper is as follows: Section 2 discusses the literature review, Section 3 explains the proposed methodology, Section 4 defines the results, and Section 5 is the conclusion of the overall paper.

## 2. Literature Review

We have divided our literature review into two sections. First, we present literature on the current and previous approaches used for PHR systems. In the second part, we describe the literature on access control model with weaknesses and strengths.

### 2.1. Literature of the State-of-the-Art Schemes

Now a days integration of blockchain with healthcare systems increase rapidly due to its distributed and non-tamper nature. The immutable features of blockchain offer a variety of application against security breaches, eves-dropping, phishing and collusion attack. Blockchain support cross-domain access control policies that is mostly widely used now a days in healthcare industries and IoT systems.

The storage of patient health record over Cloud provides various opportunities and challenges. Cloud based access control model are more proven to security breaches and a secure access control system is needed for current PHR based models. Since Cloud based framework mostly works in an open and integrated environment. Due to these features Cloud based networks are more attractive for data loss, theft, and more security attacks. Weak network security system is one of the most highlighted and explored problems which has directed IT researcher to explore more and smart security directions and tools for Cloud using medical health related data. Digital Healthcare industry has many reasons not to trust over the Cloud environment, because they cannot provide complete access control to the patient health records.The Fog Computing-based IoMT is currently a hot topic [7]. Using a health technology blockchain, Dwivedi et al. developed a peer to peer strategy for linking distant medical sensors and equipment through the Internet. They came up with the notion of a better blockchain foundation for IoT devices. In a decentralized context, this suggested approach by these authors provides higher security for a healthcare system.

Aggarwal S. et al. investigate several outstanding research topics on readers’ 5G-enabled Tactile Internet fog computing. This is also something that the researchers look into. Ahad A et al. thoroughly examine 5G-assisted smart healthcare solutions in the IoT. R. Cao et al.proposed a multi-Cloud cascade architecture, a low-overhead native testing framework, a medical data storage backup method, B.D. Deepak et al. A smart service authentication (SSA) system is proposed to improve patient-physician data security.

The impact of increased security vulnerability of electronic systems is exacerbated for devices that are part of the critical infrastructure or those used in military applications, where the likelihood of being targeted is very high.

Currently, most of the research prevails that blockchain is a promising approach to achieve security and privacy protection of personal health information. For instance, Tandon et al. [22] surveyed the benefits of integrating blockchain in healthcare to achieve security and privacy. Farouk et al.  [23] reviewed the significance of using blockchain in an IoT-enabled healthcare system for privacy-preservation that could be employed for protecting data. Turjman et al. [24] surveyed the integration of healthcare systems with blockchain for addressing health data security, integrity, ownership, privacy, and access control. Begli et al. [25] designed a secure IDS (SVM-IDS) for remote healthcare systems. Swarna et al. [26] designed a Deep Neural Network (DNN)-based IDS in an IoMT network. The underlying framework used Principal Component Analysis (PCA) and the Grey-wolf optimization technique to perform feature extraction. Newaz et al. [27] introduced HealthGuard, which uses various ML techniques to protect the healthcare system. He et al. [28] designed a DL-based IDS that uses a Stacked AE technique to secure healthcare systems. In addition, various ML techniques such as Support Vector Machine (SVM), Naive Bayes (NB), XGBoost, and k-NN were used for comparison.

**Figure 1:**
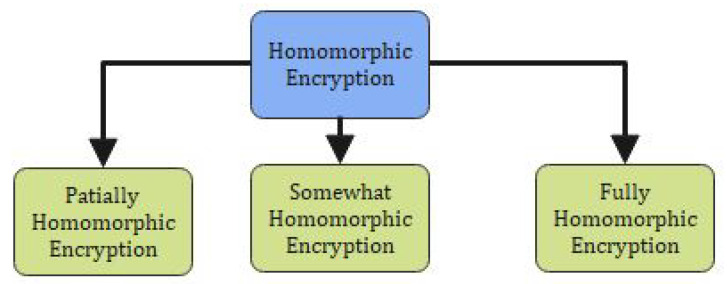
Classification of Homomorphic Encryption.

Table 1 explains the number of publications carried out using Blockchain technology in various healthcare industries [29]. From Table 1, we clearly explain different platforms and types of blockchain used in healthcare industries. In our proposed research, we will be using the hyperledger fabric platform and permissioned blockchain technology [30]. In Figure 1, the classification of the homomorphic encryption is shown and its operation.

### 2.2. Literature Review on the Methods

**Figure 2:**
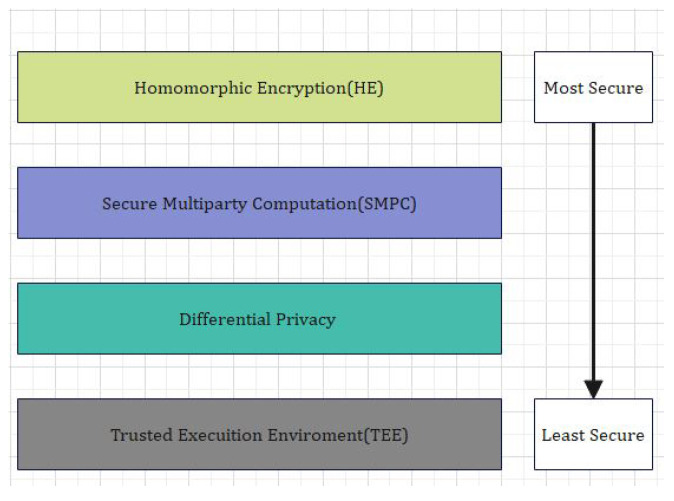
Comparative Analysis of different Privacy and Security Approaches.

To achive security using blockchain, PHR and EMR sharing has been widely applied within a decade [31]. In order to access the PHR, URLs of the PHRs are stored on the blockchain using HE techniques. The encrypted data is stored in the hospitals and healthcare institutions database which is more secure due to the HE encryption. HE technique is considered as light weight encryption techniques and hence its communication and computational cost is low as compared to ring and group signature [32]. Each digital hospital and clinic keeps PHR that creates a fractional or complete patient history to be investigated by clinicians. Digital healthcare frequently pacts with critical healthcare data about patient medical diagnosis which are more vulnerable to security breaches if would kept on centralized system [33]. There exist many common access control models based on objects, identity, role, attributes, time, domain, and trust [34]. Various cryptographic techniques have been designed and applied for access control of encrypted data. In the traditional symmetric key model, encryption is carried out using symmetric keys. The data owner divides the data into some groups and then encrypts these groups using the symmetric key [35]. Users who have the private key can decode the encrypted data. In this scheme, authorized users are listed in the ACL. The major drawback of this scheme is that the number of keys grows linearly as the number of data groups increases. In addition, if any change occurs in the user and data owner relationship, then it will affect other users in the ACL [36]. So, in summary, this scheme is not in practical use in different scenarios. Object-based models are called as DAC and MAC models. In the DAC model, an object is directly connected to the subject using the relationship between these two entities. The MAC model is the improved form of the DAC in a way that it uses the security attributes of the subject and objects to grant access. MAC (Mandatory Access Control) is considered as a standard and well established approach in cryptography. It was first designed for military purposed for controlling information. MAC indeed based on a lattice-based information flow model. MAC has two further versions, such as Bell–LaPdula and Biba models. The Bell–LaPadula model provides information flow and confidentiality, whereas the Biba model is concerned with maintaining the integrity of the data. In MAC, there is no concept of ownership. In other words, it can be described that in MAC, user rights and privileges are not resource-centric [37]. In order to explore this model more, the workings of the MAC model, partial orders, and lattices must be understood. Partial orders use mathematical sets and set properties. Partial order modeling in MAC is used to match and order resources and users’ attributes properly. Lattices are used in MAC when the information flow is almost critical. That is the main reason it was purposely designed for the defense sector. MAC uses lattices to follow the information flow policy [38]. Information flow policy deals with the flow of information from one security layer to another security layer. The flow of information can be monitored and maintained by assigning each object a security level or class. Several methods, such as machine learning and data mining techniques, rule-based models, statistical approaches, and deep-learning (DL) methods, are used to design effective security solutions [39]. DL-based IDS uses multi-layer networks of neurons that represent the mathematical computation of the learning processes [40]. The learning process of DL-based IDS depends on historical traffic data containing both benign and abnormal instances. DL-based security approaches can automatically reduce the complexity of network traffic without human involvement to identify correlations between data [41]. The BiLSTM (Bidirectional LSTM) is a useful generative models which can learn the time series data of the IIoT’ network. This training mechanism results in a DL algorithm that is powerful and fast [42]. Figure 2 illustrates the comparative analysis of different signature mechanisms and the level of security.

### 2.3. Challenges and Issues

Recently, several research works related to data privacy protection and the identification of cyber-attacks particularly using cloud and edge computing integrated with blockchain are proposed to tackle the above issues [43]. However, it should be noted that there are still various drawbacks in the system, which are listed in Table 2. The most important issue is that designing a privacy-preservation method to protect the sensitive data transactions against being accessed by unauthorized users is required. Moreover, ensuring a secured authentication data transfer scheme and maintaining data integrity when communicating the data over an IoT network is a challenging issue. Second, designing an adaptable security mechanism that can efficiently distinguish normal and attack instances in the IoT is also a challenging issue [44]. As such, the IoT network comprises various interconnected medical sensors, actuators, and machines located at multiple locations. Third, developing a new framework for deploying blockchain and deep learning techniques in current cloud-edge-assisted industrial systems is strenuous. As such framework often faces issues related to scalability, moreover, due to different computing powers of the participating edge nodes, it is infeasible to store the complete block in the edge networks [45].

## 3. Methodology

Our proposed methodology consist of the design of the framework modules, working of the system model, and the proposed algorithm. These are explained in steps in the following section. Moreover, our system model consists of three layers, namely, the IoT-enabled industrial layer, the blockchain edge layer, and the user layer. These are discussed thoroughly in the subsections of the methodology. Moreover, the proposed framework consist of the following phases in order to process a user request:

1. Registration Phase: In this phase, registration of data center full node is performed securely in offline mode by trusted registration authority. In addition, light node, i.e., sensor node, is registered using a zero knowledge proof protocol. This protocol authenticates two parties without revealing any secret identity or information. In this approach, one party becomes the challenger and the other party becomes the prover.
2. Verification Phase: In this phase, ❒P is initiated for verification from the light node end. With the receiving information from inputs, all possible values are found correct using the ❙ ❚-P and ❚❧❥ combination. The new ❙ ❚ is computed at the light node end to maintain the difficulty level by adding the value (z). The value z is computed using large prime value (❙) and corresponding generator (❚). In this scenario, it becomes the prover and (❥) becomes the verifier.
3. Validation and Block Creation Phase: If the data becomes registered, the joining of the blockchain process takes place. These are the steps involved in block creation and validation:
4. At first step, key value pair (P Bkj, P Rkj ) is created, where P Bkj is identified as a public key and P Rkj is identified as a private key of *j*th light node.
5. Furthermore, the work of registration is initiated.
6. The creates a signature and forward to respective nodes for its validation.
7. The access control policies validates the signature. Once the signature matches successfully, then the client send request of the joining network uses the credential P Bkj.
8. The security smart contracts send the validation request to the peer nodes (N-p) for validation of location of user.
9. The peer nodes (N-p) validate the location of nodes using smart contracts with timestamp recorded by smart contracts in blockchain according to the latitude and longitude.
10. Once it is verified, then True/False acknowledgment is sent back to the corresponding node.
11. For True status, a new block (Bj) is generated and appended into the blockchain network with credential P B-k-j.
12. Data generation and block updating: This phase describes the process of data creation by ($L_{N}$). The generated data are denoted as transaction (Tj). The process of data generation and the updating of the block is discussed below

### 3.1. Preliminary Data

This section comprises of what we already know and what we have about blockchain, trust, and patient health record systems. This section also describes the fundamentals of the preliminary data, research findings, and the importance of methodology.

### 3.2. Blockchain in the Healthcare System Using the Hyperledger System

**Figure 3:**
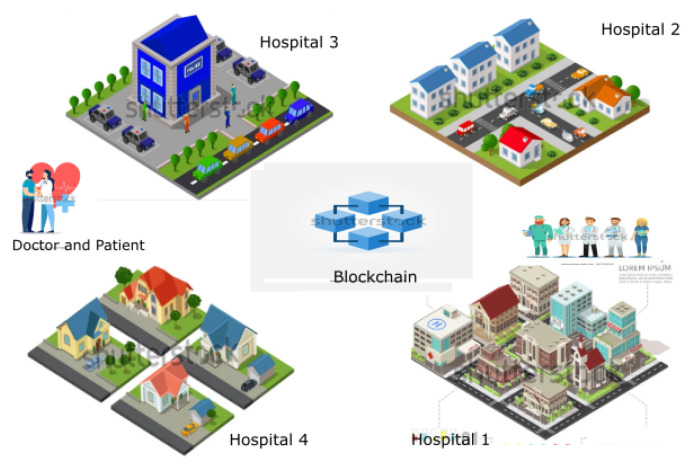
Cross-Domain Blockchain-based Healthcare System.

In Figure 2 the EHR systems is shown. From Figure 2 it is obvious that blockchain is an important tool in the field of IoT, smart cities, and SDN. For instance, Tandon et al. surveyed the benefits of integrating blockchain in healthcare to achieve security and privacy. Farouk et al. reviewed the significance of using blockchain in IoT-enabled healthcare system for privacy-preservation that could be employed for protecting data. Turjman et al. surveyed the integration of healthcare systems with blockchain for addressing health data security, integrity, ownership, privacy, and access control. Gupta et al. surveyed the advantage of smart contracts in privacy-preservation and its benefit over traditional blockchain implementation. Some research efforts are aimed at demonstrating the benefits. Performance metrics in blockchain networks, such as latency, throughput, and efficiency, have also been optimized for achieving enhanced results. Compared to traditional EHR systems, which use client-server architecture, the proposed system uses blockchain for improving efficiency and security. An IoT-enabled industrial system mainly consists of LNs, FNs, and DCs. The LNs have resource constraints and therefore can send the data to FNs in the edge-blockchain layer. The FNs can assist LNs to search transactions, and can be used for mining, and adding a new block in the blockchain network. Finally, DCs are responsible for the long term storage of data from FNs as required. The DCs sends data to the edge as per requirement [45]. The proposed blockchain-based security and privacy scheme is used to register all three nodes and therefore authenticates the data transactions in the network using proposed smart contract-based ePoW. In addition, the IPFS storage system is used to store complete transactions, and the generated hash string is stored in the blockchain [47]. Finally, the DL-based privacy and security scheme is used to transform and detect intrusions in the network [48]. Applications of blockchain includes Data distribution, redundancy, and fault tolerance  [49]. The structure and flow of data is illustrated in Figure 3.

#### Blockchain Technology and Proof of Work (PoW)

**Figure 4:**
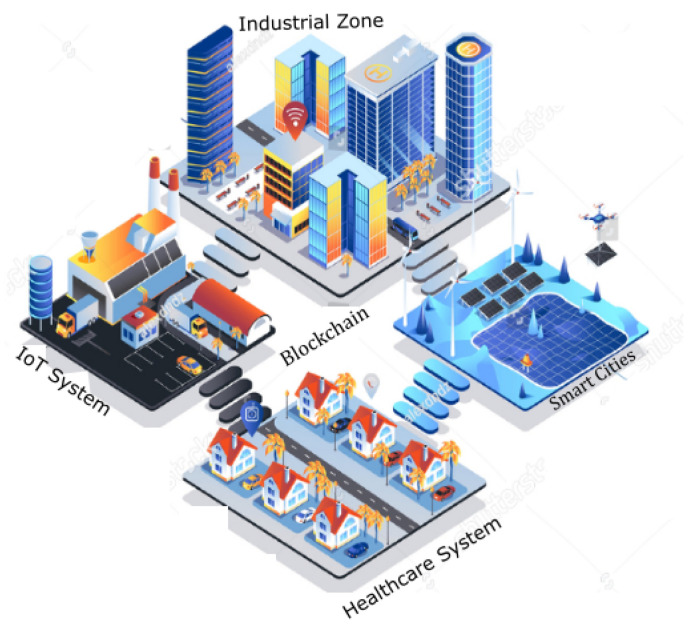
Applications of Blockchain in Various Domains.

Blockchain is an important decentralized technology used to provided peer to peer communication. The use of blockchain eliminates dependencies on centralized nodes. Transactions are approved by 51 percent of the total nodes, which is called the consensus mechanism. Blockchain is immutable and transparent technology, which means that the information or transaction stored on the blockchain cannot be tampered [50]. In Figure 4, the working of a blockchain network and its applications in various domains such as the healthcare system, the IoT, smart cities, and smart grid systems is clearly shown. Blockchain is a Peer-to-Peer (P2P) decentralized transaction ledger that could be used to secure data delivery between cloud and edge nodes in IoT [51]. The block created for a specific transaction consists of a hash of its last block, a timestamp, and related information. Once a block is added to the blockchain, it cannot be changed or tampered again; this mechanism is called proof of work (PoW). Proof of Stake (PoS) and PoW are the two common consensus algorithms used to check the validity and incorporate new blocks of a transaction inside the blockchain. However, in the entire IoT network, when a malignant miner obtains a computing power or stake greater than 51 percent, both methods could be violated; this is known as the 51% attack. In Figure 4 the details of how blockchain technology adds new nodes and peers is shown.

Between the medical devices implanted and the personal server, BAKMP-IoMT provides a secure key management mechanism. P. Gope et al. [16] presents a revolutionary anonymous Internet of Things authentication mechanism resistant to machine PUF attacks. Salem et al. [14] have developed a strategy to avoid interference with MitMs and prevent alarms from the remote health surveillance system. P. Zhang et al. [15] used a profound learning model with the deep convolutions neuronal network (CNN) and a short-term long-range memory network The approach described by Z. Ning et al. [16] can achieve a Nash balance. It’s also obtained theoretically from the algorithm’s top time complexity and the number of MEC patients. A mobile-based healthcare was proposed by Liang et al. [17]. This is also called record sharing framework using BC through an approach based on user-centric security to limit the access of unprivileged users and to enhance the privacy via channel formation scheme. The issue in this approach is the computational cost due to complex cryptographic mechanism.

Figure 4 represents the working of our proposed blockchain-based healthcare systems using intelligent smart contracts. The deep learning is implemented within the smart contracts, which predict and monitor the security threats happening from any external resources.

### 3.3. Proposed Blockchain-Based Model in IoT Using DL

**Figure 5:**
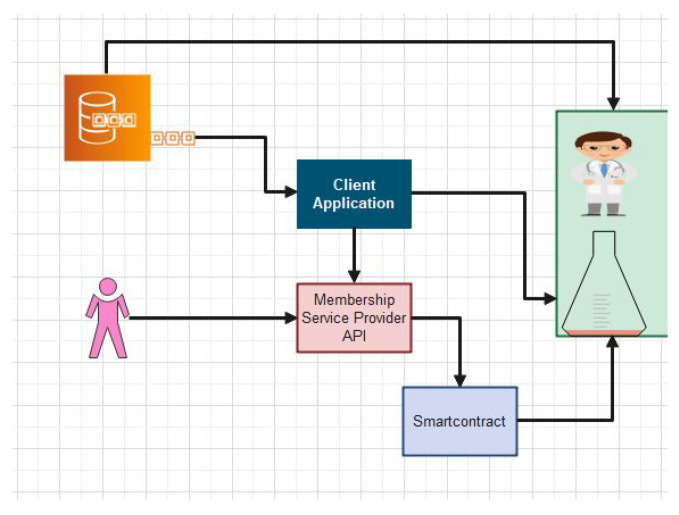
Transactions of Electronic Health Records through blockchain-based healthcare system.

The Internet of Things is the huge connection of network devices, sensors, and other electronics nodes which are interconnected. The data collected by the sensors are shared among the peers and a central device called the server. The main threats to the IoT network are its security problem and denial of service (DoS) attacks. In order to secure the IoT network, blockchain plays an important role by avoiding the central node from the network. In Figure 5, the application of the IoT in real life is shown in various domains such as healthcare, smart-cities, and sensors devices.

### 3.4. Deep Neural Networks

**Figure 6:**
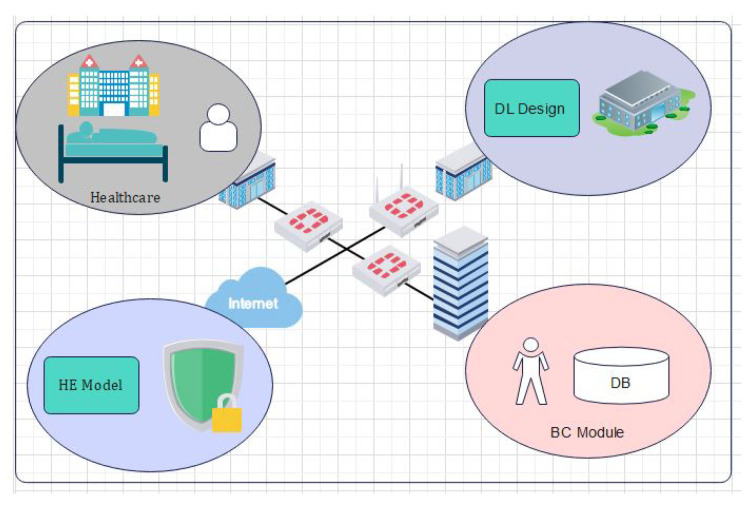
Flowchart for our Blockchain-based IoT System.

**Figure 7:**
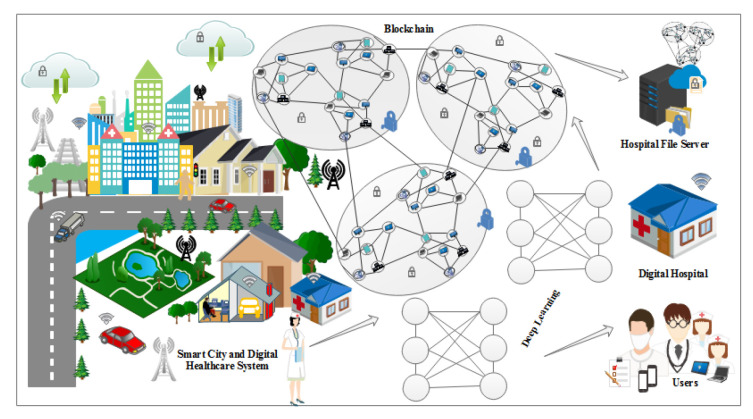
Proposed DL Module for our Blockchain-based IoT System.

Deep Neural (DL) networks play an important role in leveraging the blockchain technology’s issues and problems. Training a set of user data and behaviors provides an intelligent user-based healthcare system. We have used DL techniques in this research for tracking the exchange of public keys among mutually related parties. Our proposed techniques also provide better efficiency as compared to the benchmark models. In Figure 6, we have illustrated through diagram our proposed DL module with blockchain framework. Figure 7 provides an explanation of the deployment of our proposed framework in real life and society.

The idea of DL was taken from the human brain and brain cells called neurons. Human brains are divided into several layers, which creates a deep network. The proposed equation for DL is discussed in the equation as below. Each layer consist of its weight, and the weight is multiplied by the inputs. It can be illustrated by the following equations:

(1) $$\left[y_{i}f_{i}=f\left({(}_{i}^{n})\sum \left[xi,wi\right]\right)\right]$$

(2) $$\left[f_{x}=tanh\left(x\right)=\left[2/1+exp\left(-2x\right)\right]-\right]$$

(3) ETx(k,d)=ETx−Elec(k)+ETx−amp(k,d)

(4) ERx(k,d)=ERx−Elec(k)

(5) ERx(k)=Eelec∗k

(6) $$P^{L}\left(f\right)\propto f^{k}$$

(7) $$P^{L}\left(f,d\right)=PLo+10nlog10d/do+X\sigma$$

(8) $$P^{L}o=10log10$$

(9) $$\left(4\pi \times d\times f\right)c^{2}$$

In the equations above, the term n is the number of neurons in the hidden layer. We have used an activation function called hyperbolic tangent. The hyperbolic tangent is used in relation to the expected output. The general idea of a neural network depends on the construction of a grid made of neurons, which is composed in the form of layers. In the proposed system we have used three types of layers. The first one is called input and represented as *X*, and it takes data and processes them further. The second one is called the hidden and combined intermediate layers. The number of layers may vary, in comparison with other types. The more hidden layers or intermediate layers, the more complex the DL network will be. The intermediate layers are represented by *Y*. The last one is known as an output one, which returns the results of the classification. *The last* layer is represented by *Z*:

(10) $$p\left(e\right)=P(TgR_{e}+\vec{Vg}p_{g}\left(e-1\right)+a_{g})$$

(11) p(e)=P(T←gRe+V←gp←g(e−1)+a←g)

(12) $$h\left(m\right)=\vec{Pg}\vec{sg}\left(e\right)+O\leftarrow gs\leftarrow g\left(e\right)+ah$$

(13) h(e)=σh(→p,←p)

(14) $$logq\omega \left(j\right)=F_{K_{L}}S\omega \left(\mu |\left(j\right)\right)\left|\right|\omega \Omega \left(\mu |\left(j\right)\right)+H\delta ,\alpha \left(j\right)$$

The *h* function is used to concatenate the output sequences of neurons present in the hidden layers and can perform any four operations: add, concatenate, multiply and average. The DL technique is used in the second level of privacy. The IoT and benchmark model datasets are used to train and validate the proposed approach. This technique is used to prevent inference attacks that can become revealed from the learned model. The hyper parameters are set with the input layers from both of the datasets, and the encoder consists of two layers, including hidden nodes 50 and 25. The Relu activation and sigmoid are used as the output layer. The decoder consists of two hidden layers, including hidden nodes 50 and 25. The Relu and sigmoid functions are used as the output layers with 10 and 5 hidden nodes, respectively, for decoding the input features of the ToN-IoT, and Botnet is applied.

### 3.5. Design of Privacy-Preserving Techniques Using DL

Privacy-preservation techniques based on DL are designed as multi-layer neural networks. DL models have the ability to transform actual high-dimensional initial data into low dimensional data. This can be achieved by training multiple neural networks in such a way that the high-dimensional input data are reconstructed. DL-based architectures are of two types: generative and discriminate. Figure 7 represent our proposed three layered architecture for an intrusion detection system using DL techniques. In generative architecture, joint probability distributions are estimated using the actual distribution of each class, whereas discriminative architecture calculates the conditional probability distribution based on the data observed. AutoEncoders (AE) are the most commonly used DL-based privacy-preservation methods. A Variational AutoEncoder (VAE) technique is used in this paper compared to classical and denoising AE models. The VAE can produce new samples based on the data’s previous distribution, however, classical and denoising AEs are deterministic to features’ latent structure and cannot generate new samples. Under the above scenario, three main types of threats are relevant; information Leakage, tampering, and sabotage. The sabotage threat is not going to be addressed in this work, in practice, this risk can be mitigated, for example by enhancing the physical security of the device deployed, but cannot be completely removed, given the assumption that the adversary has physical access, so they can destroy the device or shut it down. A more serious security challenge, in this case, is information leakage. Take, for example, drones that are frequently flown into hostile territories. These devices are very likely to be shot at if discovered, which can cause financial losses. However, if a drone is captured by an adversary, this can have more dire consequences. Extracting information from such a device (e.g., origin, mission, and likely destinations) is much more valuable to the enemy than destroying it. Another threat, in this case, is false alarms, caused by noise and or environment variations. The latter may trigger undue tamper response mechanisms such as powering down the system or deletion of sensitive data, which undermines the system’s availability and disrupts its operation. Identification of threats mechanisms is explained through Algorithms 1–3. Our proposed algorithm allow only authorized users to access the EMR through Fog Computing.

### 3.6. Modeling of the Analysis Process

The analysis of previously recorded Connected network data is the second major phase in the SG inspection process. We tested the blockchain network in the simulation with various analysts operating at the same time. Every analysis must analyze Nominalise inputs. To begin, the analysts must retrieve acquisition information from the Blockchain, download a copy of the raw data electronic medical records (EMR), and validate the hash value of the EMR. The process obtains the Connects length, produces certain indicators, and runs the Add Analysis transaction if the check is successful. Lastly, the raw data EMR’s local copy is erased. is used. For secondary communication, multicasting is supported. With high-speed connection and low latency, the QoS of inter primary and primary communication increases. Fog-IoMT supports flux/ubiquitous applications, heterogeneity, and secondary connections compared to previous communications systems. Because of the distance between the components, secondary and primary communication is separated. FNs’ scalability has increased or decreased. The local connection is controlled by primary communication, while secondary communication controls the external connections. A subset of key communication is the T2T relationship. The applications were vetted and authorized on the Blockchain. To avoid congestion, a Fog-IoMT IoMT layer on a secure channel is utilized. This is also done to communicate with TFNT.

### 3.7. Proposed Intrusion Detection Method

An Intrusion Detection System (IDS) is a set of tools and mechanisms used to monitor and analyze network traffic with the aim of detecting possible intrusions (i.e., both internal and external intrusion) targeting the network. The IDS is signature- and anomaly-based. The latter detects the intrusion by matching rules/signatures against a database of their known rules, whereas anomaly-based IDS relies on interpreting normal activity to identify any deviation. Several methods such as machine learning and data mining techniques, rules-based models, statistical approaches and deep-learning (DL) methods are used to design effective security solutions. DL-based IDS uses multi-layer networks of neurons, which represents the mathematical computation of the learning processes. The learning process of DL-based IDS depends on historical traffic data containing both benign and abnormal instances. DL-based security approaches can automatically reduce the complexity of network traffic without human involvement to identify correlations between data. The bilinear LSTM (BiLSTM) is a useful generative model which can learn time series data of IoT networks. This training mechanism results in a DL algorithm that is powerful and fast.

### 3.8. Implementing Channel-Specific Smart Contracts

We have implemented our smart contracts using the proposed algorithms.

### 3.9. Deep Learning Based HE Approach

We have integrated the HE techniques with the DL in order to provide flexibility and security without decryption. ABS is an attribute-based signature with a provided signature to the attributes and transactions. We have used fully homomorphic encryption (FHE) with DL techniques, which is one of the important tools for analyzing and evaluating the activities inside a network. We have applied state-of-the-art statistical methods to evaluate these non-arithmetic functions, such as the accuracy and F1-value, with enough. Therefore, for the first time, we use the blockchain-based DL technique with the HE method in the proposed model. This approach enable us to obtain higher privacy and analyze a deep learning model on the encrypted data. We mathematically and through simulations verified that the proposed model with the IoT-Botnet dataset shows 98.67% results to the original IoT-based Blockchain model with non-encrypted data. The classification accuracy of the proposed model is 90.6%, which is quite similar to that of the CNN model.

Each algorithm is described in the below sections:

### 3.10. Proposed Secure Search Algorithm

We have designed a novel secure searchable algorithm that provides the facility to the users to encrypt at their one side and upload it to the distributed ledger. Through our proposed extended secure searchable algorithm user can anonymously search the keywords using blockchain users API. In case the user lost the key, he or she can revoke the policy and can request a new key. It provides protection against active collusion attacks.

Algorithm 1 describes the working of the Algorithm 1 and the flow of attribute signing techniques that we proposed for the transactions and encryption of the participants attributes.

**Figure 8:**
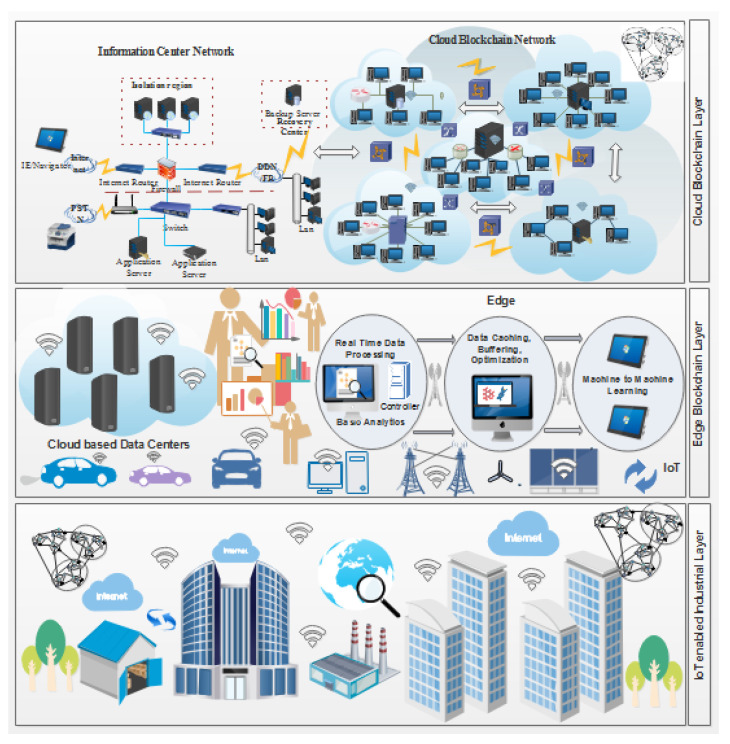
Proposed Deep Learning-based Intrusion detection model for our blockchain framework.

Our proposed framework consist of four main participants, i.e., admin, doctor, patient, and lab technician. We have proposed delegation policies and algorithms for each node. Our proposed framework is illustrated through Figure 8. A patient registers to a hospital using the blockchain API (Application Programming Interface). Doctors and patients can store, retrieve, and update electronic health records (EHR) in the blockchain repository through smart contracts. We have proposed a novel secure search-able algorithm which provides the facility to the users to search the encrypted keywords and access it without decryption. Access control policies are embedded in smart contracts, which check the level of integrity and security of any users who want to access the EHR. Users’ behaviors and activities are monitored and classified through DL techniques.
**Algorithm 1** Attribute Based Signing Algorithm**Input**: Initiate Master public key Ppub-s of domain, system parameters of domain, message M_0_, e’s identity I_*De*_, and digital signature (h_0_, S_0_) **Output**: Result of verification: pass or fail
  1:Convert the Value of h_0_ to int  2:if h_0_ ∈ [1, N × 1] Not ≤, the verif fails  3:Compt Value t = g h_0_ in G^*T*^  4:Compt ω= H_2_(h|δ, N)  5:Compt δ = (r × h) mod N; if l = 0, move to sage 2)  6:Compt α = H1(IDe||hid, N)  7:Compt Value P = [h_1_]P_2_ + P_pub-s_ in G2  8:Compt Value u = e(S_0_, P) in G^*T*^  9:Compt w_0_ = u · t in G^*T*^10:convt the Value of w_0_ to a bit string11:Compt int h_2_ = H_2_(M_0_||w_0_, N)12:if h_2_ = h_0_ holds, the verification13:Otherwise, the verification fails14:End Compt15:Ret O16:End Procedure

**Algorithm 2** Algorithm Method Evaluation
  1:Enhance Manifold Analysis Evaluation of both the IoMT end  2:SelectIoMT device for comm  3:Get acquisition, hash, electronic medical records (EMR)  4:Extract EMRFromRepository from EMR (EMR name)  5:EMR, valid SHA256 checkHash (EMR, hash)  6:if EMR, valid is true, then  7:Get the Connect Length using Connect length (Connect)  8:Generate Indications(Connect length) Generate Indications(Connect length)  9:F Blockchain transaction addAnalysis(i, indications)10:deleteLocalEMR11:end if (EMR)12:end13:end


**Algorithm 3** Access Control Algorithm**Input**: Public Key. **Output**: Verification result: succeed or fail
  1:Write r ∈ [1, N × 1]  2:Calculate ω = g^r^ in G_*T*_  3:Convrt the data type ω into a bit string  4:Compt int h = H_2_(M||w, N)  5:Compt int l = (r × h) mod N  6:if l = 0, go to step 2  7:Compt value α = [l] ske in G1  8:Convrt value h and S to a byte string, output (h,S)  9:END If10:End Procedure’s

## 4. Proposed Access Control System for Framework

**Figure 9:**
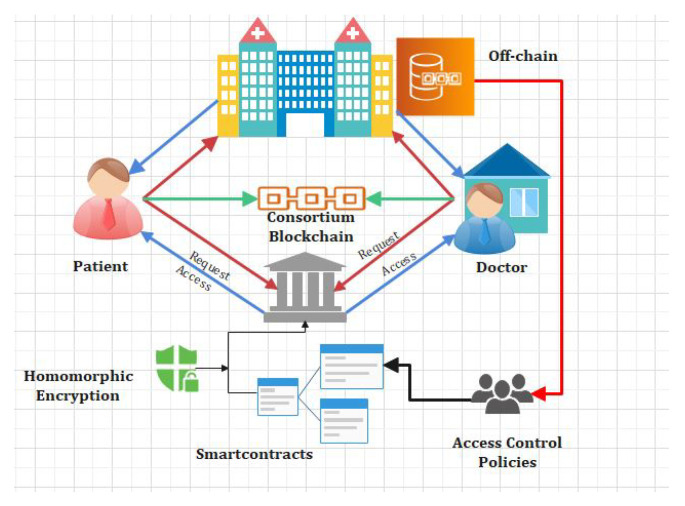
Keyword Search based Framework for EHR System.

In this section, the complete details about the cross-domain permissioned blockchain framework are explained. Figure 8 and Figure 9 represent the detailed structure of the proposed framework. The framework includes three layers. Herein, Layer 1 is known as the user-level layer, that is responsible for interface with a patient, medical practitoners and doctors. Layer 2 is called the underlay layer. Its functions is to connect the local network of hospitals. The framework is based on four novel algorithms. Each algorithm function is implemented in smart contract. These algorithms are explained in the next section. In the suggested framework, layer three is also known as an overlay network. In other words, we refer to it as a cross domain network of blockchain. Homomorphic encryption and its working is represented through Figure 8. In order to search keywords securely, as well as anonymously, we have used the application of a novel approach called homomorphic encryption. Two main functions are used in the process of cryptography, i.e., encryption and decryption. We have justified that our proposed model is more secure through experimental analysis presented under the results section.

### Proposed Algorithms

**Figure 10:**
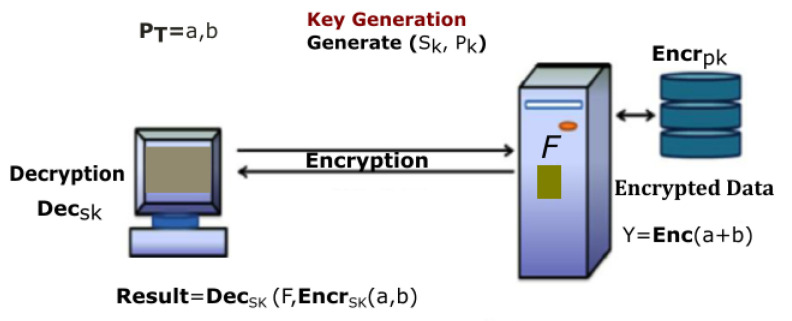
Trapdoor Generation Time using Homomorphic encryption based keyword search.

The proposed algorithms are explained through psudocode and it explains the working of the smartcontracts for the proposed system. The working of blockchain and its working for admin module in a blockchain network is shown in Algorithm 1. The user requests an enrollment certificate through the admin nodes. The admin has full rights to the access control policies, which includes write, read, update, and removal of participants. The admin node can delete a user from the blockchain list in case of revoking the access control policies. When a participant’s request for accessing the EHR or PHR smart contracts inside the blockchain is triggered, the security smartcontracts is triggered and checks for the eligibility. If the participant has enough valid attributes, then access is granted, otherwise, it is denied. The DL techniques monitor the behavior. The proposed policies monitor the activties of the user and if it is found to be malicious, then admin remove it from the system. Table 1 lists all acronyms used in the algorithm. The systematic execution of the patient module is shown in Algorithm 3. Algorithm 3 is based on a secure keyword search as shown through code. This procedure for the patient node uses its identification to the blockchain network. Our proposed Algorithm 3 is based on secure access control. If a participant requests to access a session or update a record, Algorithm 2 validates the attributes of the requester. Moreover, these attributes comprised of name, ID, social security number, location, address, gender, age, organization, marital status, and other important attributes. The proposed algorithm provides a more secure privacy and access to the users records. However, Algorithm 3 is more based on fine-grained access control. In other words, this algorithm provides a secure key for each request and each record individually. The proposed approach leads to granular access control and privacy methods. Therefore, our proposed system provides more validity and security. Figure 10 illustrates the implementation and working of proposed smart contracts. The access control policies are implemented through smart contracts. The working of each smart contract is clearly mentioned and explained through pictorial view in Figure 10.

## 5. Experimental Results and Discussion

This section is comprised of experimental setup, simulations results, and discussions as below:

### 5.1. Experimental Setup

Authentication methods for users differ widely from those for computers [29], which can perform gigabytes of computations within a twinkle of an eye that humans cannot. For instance, the cryptographic protocols such as homomorphic encryption shall lose viability if used for authenticating humans, for that matter, the methods. In the proposed framework, the blockchain architecture was developed using Hyperledger Fabric version 1.1.2 and Solidity (version 6.0). The deep-learning architecture was designed using the Python library Keras. The system was configured according to the hardware and software requirement of blockchain. The performance of the proposed system for intrusion detection was conducted using IoT data sets. Both data sets were split into 70 percent and 30 percent training and testing sets, respectively [28].

### 5.2. Dataset

The initial dataset is presented with all the information from the log file described above. By making a split for each space in each log row, it results in ten columns. After inputting the log file and extrapolating a schema, only the columns needed for future training were selected. In particular, the IP address is essential to differentiate the requests from which client they were made and then create the label to differentiate licit users from illicit ones; the date is important to create the 30 s periods necessary to the model to perform the training; and lastly, the size of the request bytes and also the only one to give input to the neural network. The final dataset is structured in such a way as to have periods of 30 steps, each regarding the sum of requests made by an IP address in a small range of time, with three columns: IP, Byte and Label; the latter identifies the type of user (licit, illicit). The IP column has not been removed because for test data in a real environment, where you will not know if it is a licit or illicit request, once the prediction has been made you will need to identify the attacker in case a DDoS attack is in progress. The Information exchange the target application comprises a large amount of healthcare device data, such as IoMT, which is rapidly growing. There is a need for more bandwidth, data storage, and capacity. It sends data to and from local storage devices, online devices, and the Internet. The data is remediated, filtered, and merged under corporate standards. In this case, the Cloud is used as the final layer for metadata processing. Data and metadata analysis are summarized by IoT nodes (Edge or Dew Computing (DC)). With IoT nodes and dew computing (DC), the proposed Fog-IoMT Architecture improves the mobility of IoE (Internet of everything) users. Blockchain (BC) adds a second layer of security to prevent anonymous users from using IoMT devices. The following is the order in which the communication takes place: (a) Wireless Internet access is used to communicate with IoMT devices across a medium distance. TCP/IP is utilized for inter-primary communication, whereas ZigBee and Bluetooth are used for primary communication. (b) Wireless or wired media communicate between Cloud computing, dew computers, and the fog nodes. TCP/IP end-to-end connections are made via CAT-5/6 optical fiber. There are two sorts of communication: direct and indirect.

### 5.3. Results Analysis

**Figure 11:**
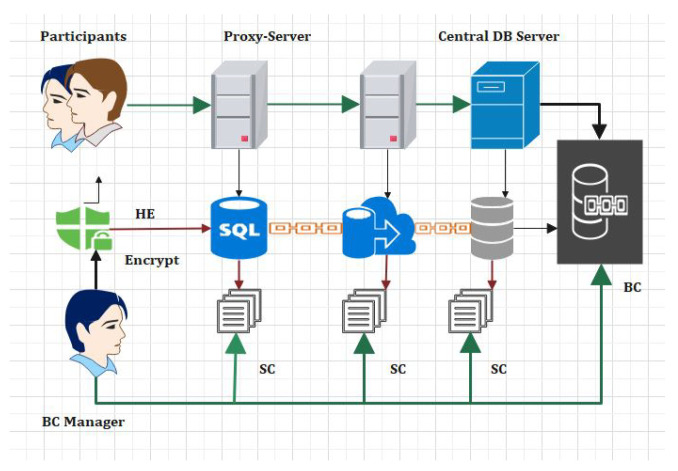
Working of Proposed Secure searchable framework.

**Figure 12:**
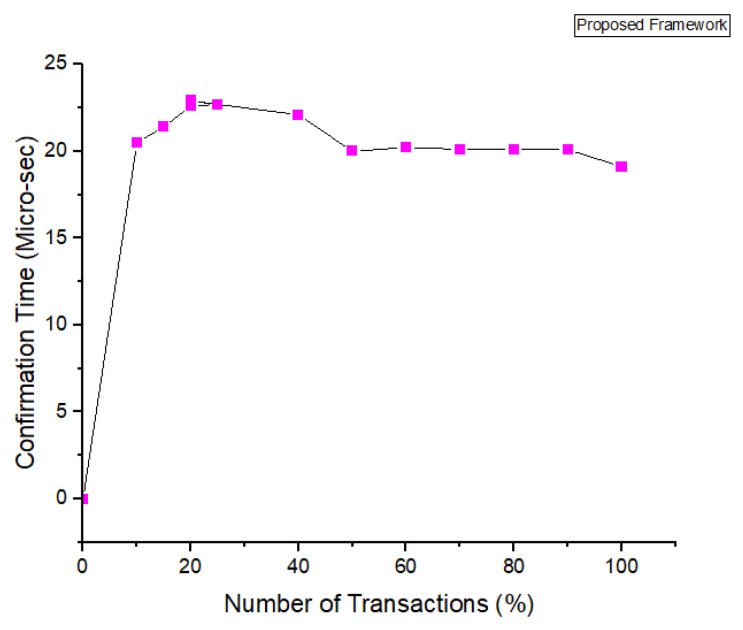
Simulations Results for proposed Method-Confirmation Time vs. no. Transactions.

**Figure 13:**
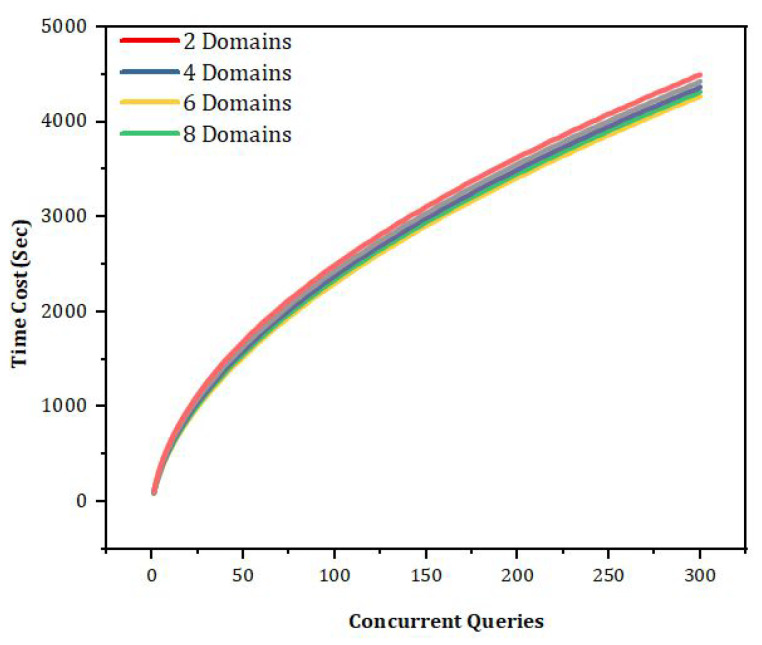
The impact of various domains over the time cost and number of transaction based on number of concurrent queries and requests.

**Figure 14:**
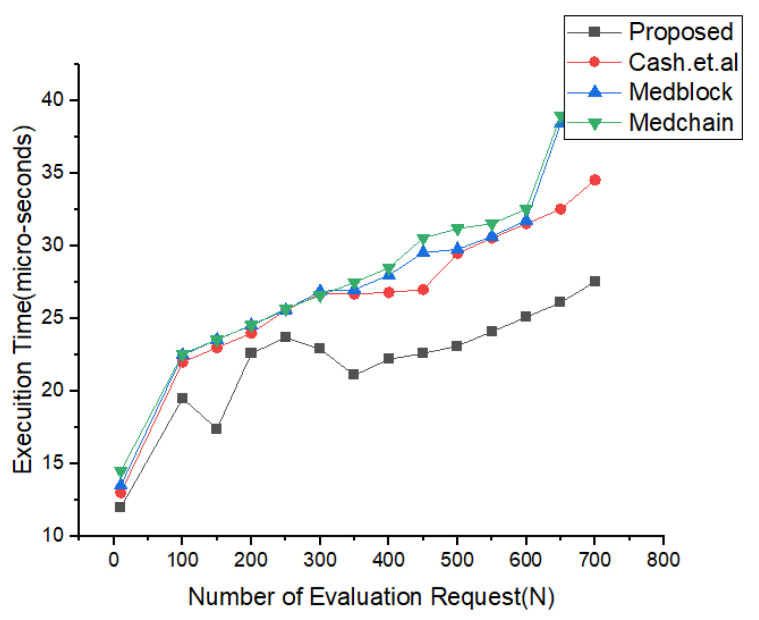
Comparative Analysis of Access Control Models.

**Figure 15:**
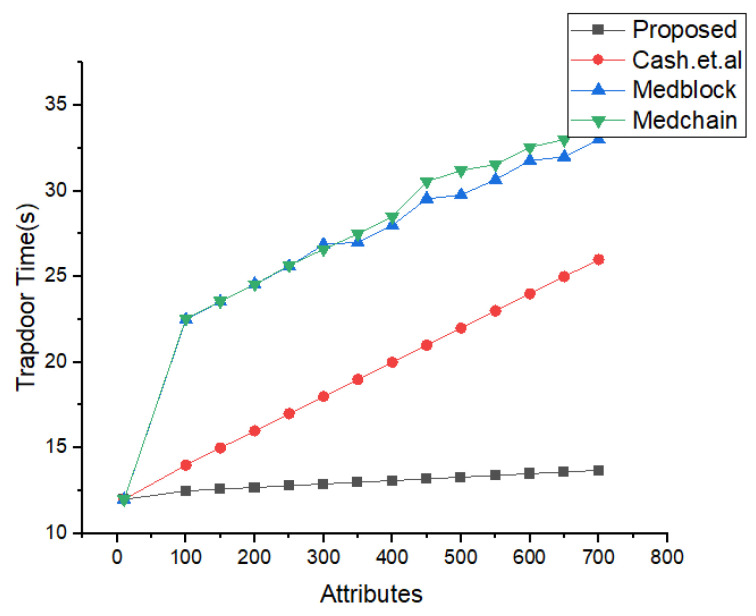
Trapdoor Generation Time using Homomorphic encryption-based keyword search.

**Figure 16:**
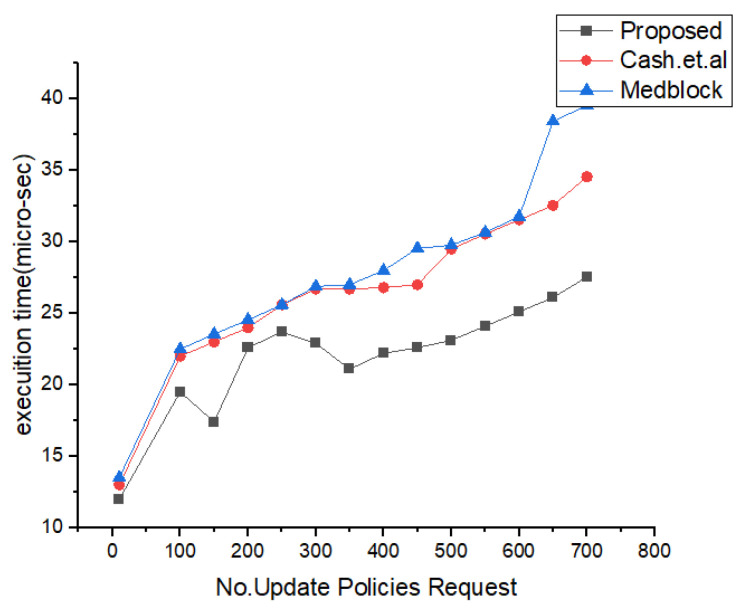
Experimental results for Proposed Method vs. benchmark Models.

**Figure 17:**
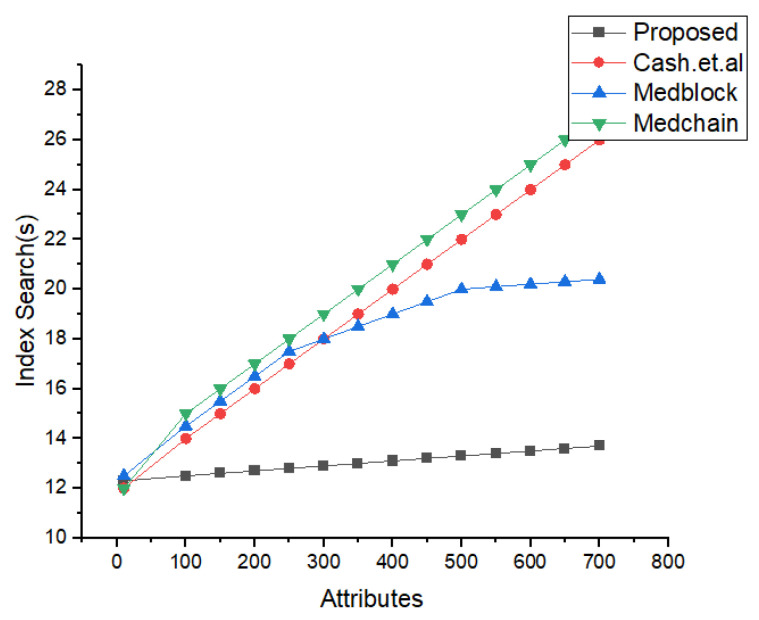
Index Search Based Comparative Analysis.

**Figure 18:**
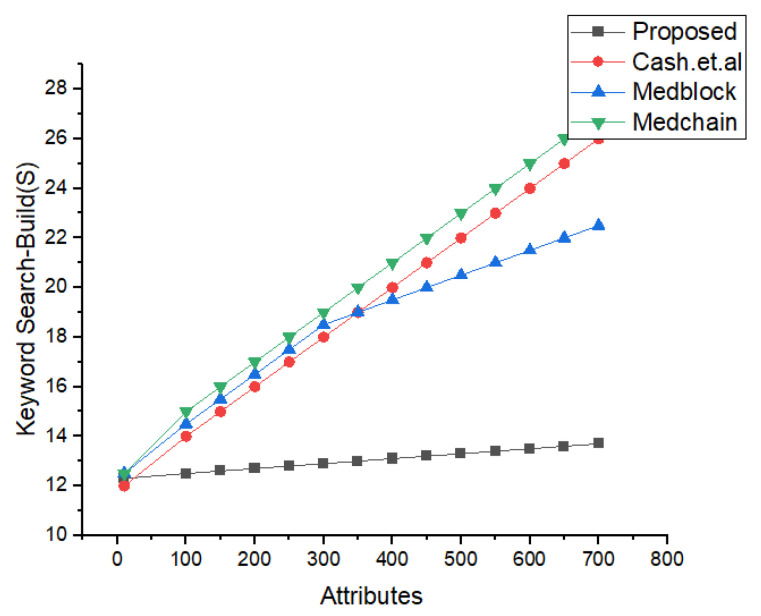
Comparative Analysis of Search time vs. attributes.

**Figure 19:**
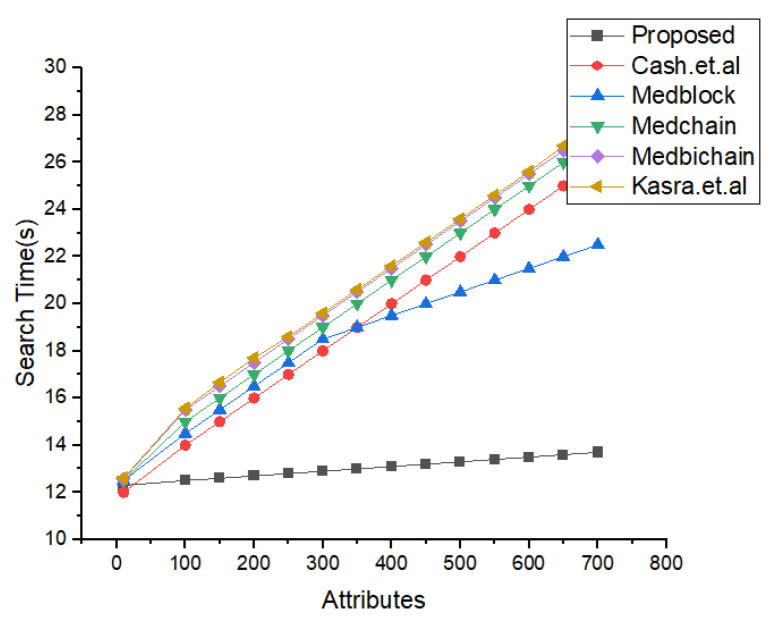
Comparative Analysis of concurrent requests for the proposed policies.

**Figure 20:**
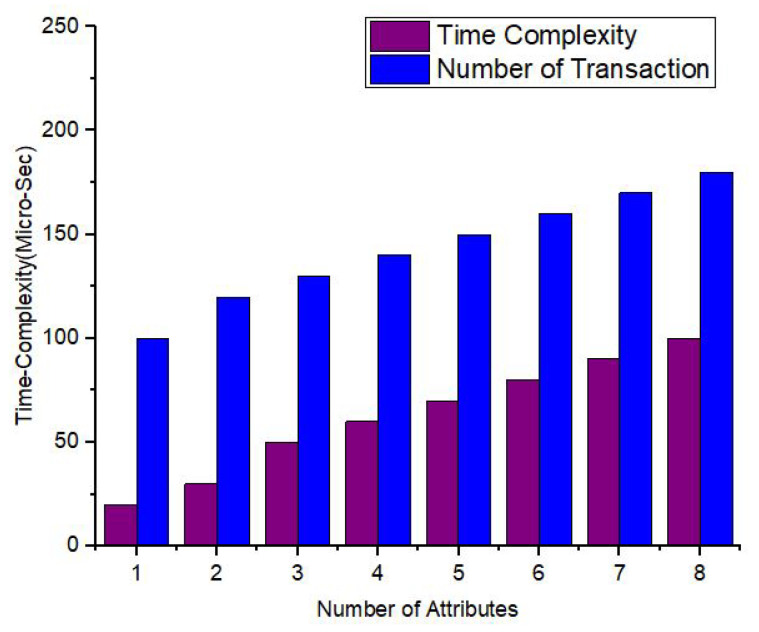
Comparative Analysis of Cross-domain using Homomorphic Encryption.

**Figure 21:**
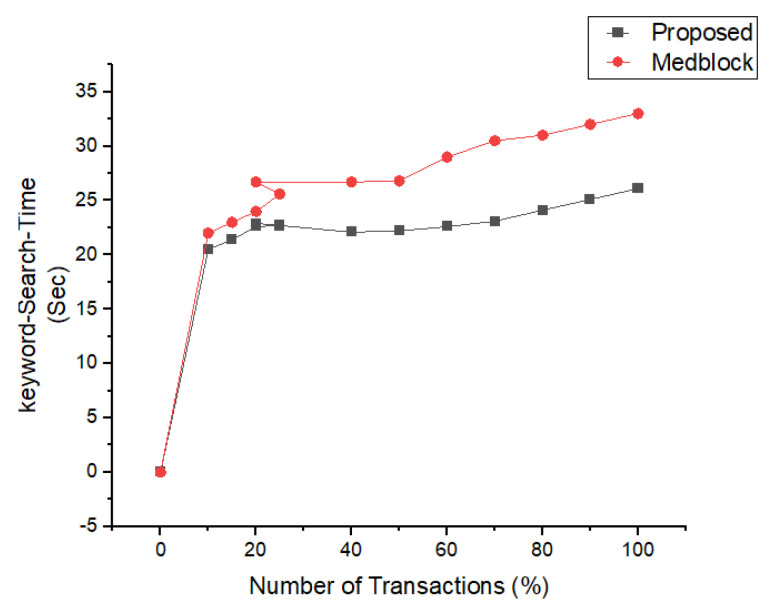
Comparative Analysis of keyword Search Mechanism in proposed and Benchmark Model.

**Figure 22:**
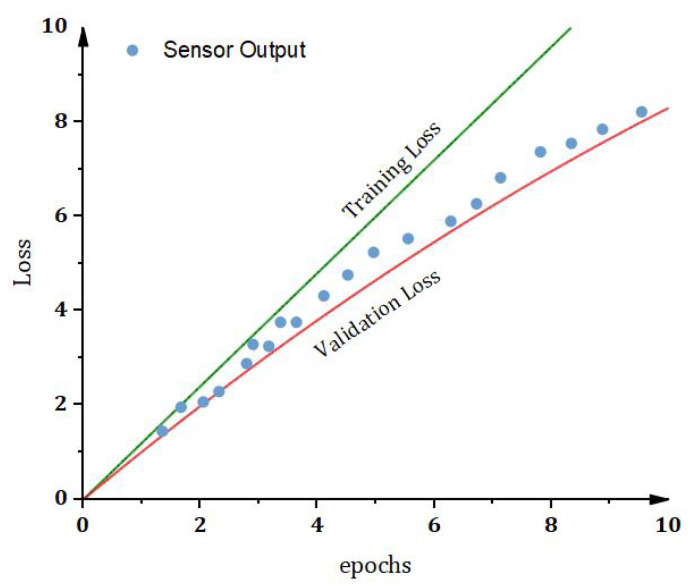
The Accuracy vs. loss for validating the proposed method using IoT dataset.

In this section we have carried out analysis of the proposed framework and the eperimental work. Moreover, we have also carried out analysis based on those parameters that can effect positively the proposed framework. The below figures show the values obtained with proposed concept. The result parameters with respect to accuracy (acc) and loss shows that our proposed model has efficiently learned from both data sets, as illustrated in Figure 10, Figure 11, Figure 12, Figure 13, Figure 14, Figure 15, Figure 16, Figure 17, Figure 18, Figure 19, Figure 20, Figure 21 and Figure 22. The proposed model has accomplished about 94.34 percent accuracy and 8.89 loss with the ToN data set, while the IoT model obtains 88.38 percent accuracy and 8.92 loss. The purpose of the proposed model is not to detect these malicious observations but to encode the data into new dimensions. This transformed data are further used in designing an efficient IDS with high system performance. The security analysis of the proposed model is performed using Automated Validation of Internet Security Protocols and Applications (AVISPA) software validation tool. In our proposed research, Hyperledger caliper will be used as a tool for the blockchain network. It has the ability to support different hyperledger frameworks, e.g., fabric, composer, sawtooth, iroha, etc. We have implemented the homomorphic encryption for our encryption and decryption in order to provide secure searchable encryption mechanism. In this proposed research, the caliper tool plays an important role in the verification and execution of the system, as well as various parameters. The parameters includes latency, throughput, encryption and decryption time, and computational cost. Our simulation setup configurations consist of the following specifications:

Experiment 1: We run our first experiment up to 800 rounds, and we evaluated our results based on the number of the personal health records sent versus the number of rounds. Experiment 2: We run our experiment 2 for 400 numbers of rounds we evaluated the efficiency of the proposed system according to the number of PHR sent versus number of rounds or transactions.

In Figure 9, we plot the results for the number of rounds taken and the number of transactions sent per second are highlighted. From the simulations, we can see that our proposed framework is much better than the benchmark models. We have achieved more efficiency as compared to the benchmark models. In Figure 10 we have provided the analysis of the proposed framework using homomorphic encryption techniques. Through our proposed framework, we have evaluated the encryption based on each keyword search and the number of attributes selected to achieve the maximum throughput. We observed the execution time and the number of policies for each attributes. In Figure 10, we have provided the simulations results for the number of transaction sent through the permissioned blockchain and the confirmation time measured in seconds. This is supported by Algorithm 1. We have discussed the Algorithm 1 in complete details for the transaction. The algorithm takes the input request from the users and transfers the transaction through smart contracts. Each transaction posses communication and complexity. We have provided the simulation results in Figure 10. The syntax for these simulations results are mentioned through our keyword search and homomorphic encryption algorithms. Through our proposed framework, users can encrypt data on their own side and upload them to the blockchain.

In Figure 11, we have illustrated the simulations results and the analysis of our proposed framework versus the execution time. From the simulations in Figure 11, it is very clear that our proposed framework provide more efficiency as compared to the benchmark models. It can be easily seen that the authorization policy took less time as compared to the authentication policy and delegation policy. These simulations in Figure 12 justify that the our proposed access control policy provides more security and less computational cost. Figure 13 describes the simulation results of the number of users classified based on their interaction and behavior with the proposed framework. Figure 12 illustrates the details of the simulations for the trapdoor and the number of attributes. We have compared our proposed framework with the benchmark models using the same data sets and same number of attributes. We evaluated in these experiments the number of rounds as the input and the number of packets sent to the cluster as the output. From these simulations, it is very clear that we have achieved the maximum efficiency and throughput for the same data set used in the literature, i.e., Medrec and Medblock.

In Figure 13, the simulation results for the searching time and the number of attributes are shown. We have compared the proposed research method of the search time and the attributes with the latest benchmark models based on homomorphic encryption and the healthcare system. From the simulations’ results, its very clear that the proposed framework performs better than the benchmark models for the same number of parameters. The algorithm for the index searching is describe in our proposed algorithm for index searching techniques. The benchmark model’s communication and search times are greater compared to the proposed framework.

In Figure 14, we have explained the comparative analysis of the proposed methodology and the benchmark methods. For the same data set, we run our experiments in our proposed framework, and we compared the efficiency and number of packet sent to the nodes. It can be easily observed that the number of patient health records sent to the base station are more in number in the case of our proposed framework. In the case of benchmark-based models, it can be seen that it is very less comparatively. We have improved the efficiency 1.9 times as compared to the benchmark models. Table 3 provides the predictive value based on the proposed techniques, i.e., homomorphic encryption and we have predicted the number of false positives and false negatives. In addition, from Table 3 it is very obvious that our proposed method provides more accuracy as compared to the previous benchmark models. In Figure 15 we have described the comparative analysis of the concurrent request for our proposed policies. Our policies support the concurrent request. However, these requests are not limited to read or write; they also consist of update and delete policies.

In Figure 16, the simulation results based on the homomorphic encryption for keyword search. From the results, it can be concluded that our proposed method support cross-domain as well ad fine-grained access control approach. The proposed model resist the tampering, security breaches and unauthenticated access. In Figure 17, we have explained the number of transaction sent from one domain to another domain. It can be easily observed that the number of transactions which mean the number of patient health records (PHR) or electronic health records (EHR) sent per round. We run our simulations for 800 rounds and evaluated the number of patient health records sent. We did comparative analysis with the benchmark models such as Medrec and Medblock.

In Figure 18, we have described the experimental results for our keyword search mechanism in our proposed framework and benchmark models. Our benchmark models consist of the Cash et al., Kasra et al., and Medblock and Medchain. We can see from the simulations results that our proposed framework performs better than the benchmark models in searching the encrypted keyword. Our proposed method provides less communication cost for each keyword search in the blockchain framework. Our method is also valid for the cross-domain.

In Figure 19, we have described the simulation results based on our proposed policies. We have proposed access control policies for our proposed framework using homomorphic encryption and pseudo random algorithms. We have evaluated our proposed access control policies against the number of execution time and number of access policies. We did experiments on the policy revocation, policy creation, and add policy.

Simulation results in Figure 20 provide the illustrations of experimental analysis of number of transactions based on number of nodes and the execution time. Its very clear from the simulations results that the number of transactions up to 250 takes less execution time for the less number of nodes as compared to a greater number of nodes. The result parameters with respect to accuracy (acc) and loss shows that our proposed technique has efficiently learned from both datasets, as illustrated in Figure 18 and Figure 19. The proposed model has accomplished about 94.34 percent acc and 8.89 loss with the IoT dataset, while the IoT-Botnet model obtains 88.38 percent acc and 8.92 loss.

The proposed framework and algorithm purpose is not to detect the unusual activities with the system coming from outside. The proposed system detect the unfriendly and unusual behavior of the users through the implemented algorithms which are based on smartcontracts using homomorphic encryption.

In order to ensure the security and privacy in proposed framework, each IoT node is assumed to be enrolled with the proposed system model. The process of enrollment is shown in Figure 11. The Figure 12 describes the actual IoT sensor data upload time with consortium blockchain secured storage layer with number of transactions. In the proposed framework the number of transaction depends the number of nodes and the encryption techniques. We have used lightweight encryption system which is called homomorphic encryption and through this the proposed model can transfer more transaction within the required time.

**Figure 23:**
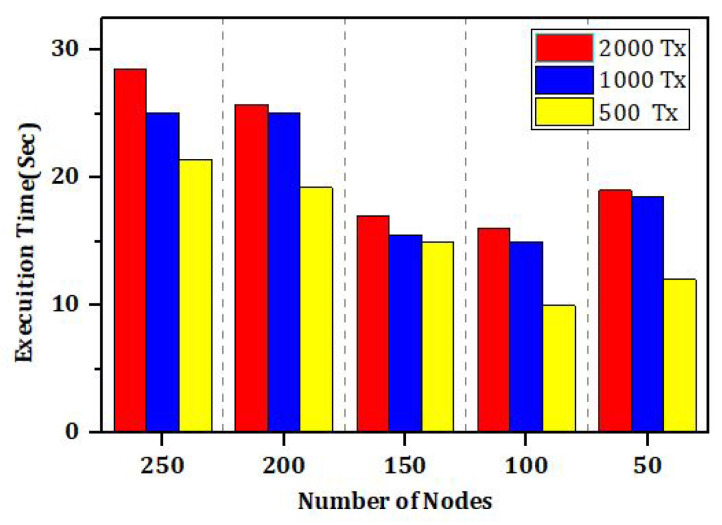
The impact of number of nodes on execution time and number of Transactions (Tx).

In Figure 19, Figure 20 and Figure 21, we have simulated the block mining time, number of rounds, umber of transactions, and the search time for each keyword. It can be easily seen that the time increases as the number of IoT sensor nodes increase. However, in our proposed framework we have used lightweight encryption technique and smartcontracts that take less execution time as well as search time, hence reduce the communication cost. The Figure 22 illustrates the number of transactions sent by each group of nodes. The comparison is carried out on the number of IoT sensor data transfers from the source node to the destination node through the blockchain framework using DL-based smart contracts.The transaction sign ensures non-repudiation in the framework proposed. Table 4 provides the conclusion of our proposed model and the benchmark. Figure 23 shows the nodes impact on transactions number and time of execution.

The result parameters with respect to accuracy (acc) and loss shows that our proposed method has efficiently learned from both data sets, as illustrated in Figure 19, Figure 20, Figure 21 and Figure 22. The proposed model has accomplished about 94.34 percent acc and 8.89 loss using IoT dataset, while the benchmark model obtains 88.38 percent acc and 8.92 loss. The purpose is not to detect these malicious observations but to encode the data into new dimensions. This transformed data are further used in designing an efficient IDS with high system performance.

Furthermore, the propose privacy method is evaluated as the smartcontracts based on our proposed model. The hyperparameters are configured by an input layer fed from both datasets and 5 hidden layers, with hidden nodes = 100, 75, 50, 25, and 15, respectively, a Relu activation function, and the output layer includes a Softmax activation function. The system configuration with loss = categorical cross-entropy, optimizer = N, epochs = 20, and batch size = 50. The simulations results are achieved before and after applying two-level privacy-preservation technique. The proposed model with the transformed IoT dataset has achieved 0.0167 loss and 99.58 acc, while 0.0052 loss and 99.89 acc with the actual dataset. Similarly, the transformed benchmark dataset model has obtained 5.5116 loss and 90.86 acc, while 0.0685 loss and 99.98 acc with the actual dataset.

Table 4 provides the overall comparative analysis of our proposed framework and the benchmark models. We also evaluate the proposed model in terms of class wise prediction results, i.e., PR (Prediction Ratio), DR (Detection Rate), F1 (Acc) and FAR (False Acceptance Ratio) metrics.

These metrics are based on True/False values. In Table 3, we see that the model with actual and transformed IoT data sets has achieved in an average of 90 percent–100 percent values for PR, DR, and F1 score and has reduced FAR close to 0 percent. Similarly, in Table 4, for various types of attacks such as DoS, DDoS, Reconnaissance, and Normal group of actual IoT-Botnet data set model has achieved in an average of 99 percent–100 percent values for PR, DR, and F1 metrics.

## 6. Conclusions and Future Works

In this paper, we have implemented a novel extended approach of homomorphic encryption in a digital healthcare system leveraging blockchain technology and DL, which provides secure keyword search facility at the user end. Our proposed scheme supports immutable, tamper resistant, and delivery secured data, which results in the reduction of security breaches to the healthcare data. The deep learning technique has been introduced to train the model, which can predict and monitor the attacks such as DoS, DDoS, and collusion resistance. For training and classification, we have divided our data set into two categories, i.e., training and testing data sets, where 70 percent of the data set has been used for training and 30 percent for testing purposes for cross-validation. Furthermore, our novel mechanism allows blockchain users to encrypt data at their own side and upload it to the distributed ledger for record purpose. Users can securely search the desired health-related data without decryption based on homomorphic SSE. We have provided a comparison to the benchmark models such as [13,30,50,51,52]. Our proposed approach is resistant to active collusion and replay attacks due to the flexible policy revocation. Blockchain technology also supports distributed data, redundancy, and fault tolerance features for digital systems. Following the proposed research, current challenges and problems in the literature faced by the digital healthcare industry were solved. More and more, we proposed a framework and algorithms that enables access control policy for users to achieve privacy and security for patient health data in the PHR system. The proposed method provides more independence to the the users, and it supports flexibility and fine-grained keyword searches. We have justified our proposed research algorithms and polices through simulations run on hyperldegr fabric tool. We used the Pycharm tool for data analysis. With our proposed method as the most up-to-date approach applied first on healthcare and blockchain technology. We have improved the security and anonymity as compared to the benchmark models such as Medrec, Medchain, and Medbichain. In the future, the proposed model can be enhanced by applying different deep learning techniques such as classification methods. 

## Figures and Tables

**Table 1:** List of Proposed Acronym used.

List of Acronyms	Explanation
DAC	Discretionary Access Control
MAC	Mandatory Access Control
ABE	Attribute Based Encryption
ABAC	Attribute Based Access Control
N	Number of Transactions
Dn	Dead Nodes
DL	Deep Learning
HE	Homomorphic Encryption
BC	Blockchain
PHR	Personal Health Records

**Table 2:** Access control type, scope, scale, privacy issues, real time data set used, and accuracies of various occupancy techniques.

Technique/Technology	Reference	Scope (Shape/Size)	Scale (Number of People)	Privacy Issues	Sampling Time	Accuracy
Access Control	[1]	NA	18	Yes	Yes	80%
	[26]	60	NA	Yes	Yes	80%
	[8]	250	NA	Yes	Yes	80%
	[10]	100	NA	Yes	NA	92%
	[28]	100	8	Yes	Yes	NA
Access Control Types	[23]	50	1	No	Yes	NA
	[3]	NA	1	No	NA	NA
	[28]	NA	14	No	yes	86%
	[9]	NA	1	No	Yes	75%
Framework	[32]	100	2	No	yes	NA
	[10]	NA	150	No	NA	90%
Security	[11]	50	1	Yes	yes	93%
	[18]	200	NA	Yes	yes	79%
	[15]	100	NA	Yes	No s	NA
	[17]	50	6	Yes	No s	60%
	[21]	100	30	Yes	NA	91%
Data Storage	[20]	NA	45	Yes	Yes	70%
	[14]	NA	4	No	Yes	80%
	[18]	100	4	No	Yes	NA
	[43]	40	9	No	Yes	80%
	[19]	200	23	No	NA	NA
	[26]	100	1	No	NA	70%
	[25]	50	3	No	Yes	80%
	[32]	150	3	No	Yes	85%
	[34]	NA	3	No	Yes	73%
Efficiency	[12]	NA	72	No	NA	55%
	[46]	100	41	No	No	86%
	[22]	200	10	No	No	NA
	[46]	NA	NA	No	No	91%

**Table 3:** List of Proposed Parameters.

Simulations Parameters	Explanation
Attrb	Attributes
B_*s*_	Block Search
K_*s*_	Keyword Search
I_*s*_	Index Search
N	Number of Transactions
HW	GPU enable Hardware
DN	Dead Nodes
CH	Cluster Head
HE	Homomorphic Encryption
BC	Blockchain
SC	Smart contracts

**Table 4:** Attack Resistance based on Secure search Mechanism.

Models	Collusion Attacks	DoS	DDoS
Medblock	No	No	Yes
Casht et al.	Yes	No	No
Medchain	Yes	No	No
Kasra et al.	Yes	No	No
Proposed	Yes	Yes	Yes

## Data Availability

The study did not report any data.
